# Human Health Impact of Cross-Connections in Non-Potable Reuse Systems

**DOI:** 10.3390/w10101352

**Published:** 2018

**Authors:** Mary E. Schoen, Michael A. Jahne, Jay L. Garland

**Affiliations:** 1 Soller Environmental, Inc., 3022 King St., Berkeley, CA 94703, USA; 2 U.S. Environmental Protection Agency, 26 W. Martin Luther King Drive, Cincinnati, OH 45268, USA

**Keywords:** QMRA, cross-connection, onsite, non-potable, reclaimed, greywater, wastewater

## Abstract

We used quantitative microbial risk assessment (QMRA) to estimate the microbial risks from two contamination pathways in onsite non-potable water systems (ONWS): contamination of potable water by (treated) reclaimed, non-potable water and contamination of reclaimed, non-potable water by wastewater or greywater. A range of system sizes, event durations, fraction of users exposed, and intrusion dilutions were considered (chlorine residual disinfection was not included). The predicted annual microbial infection risk from domestic, non-potable reuse remained below the selected benchmark given isolated, short-duration intrusion (i.e., 5-day) events of reclaimed water in potable water. Whereas, intrusions of wastewater into reclaimed, non-potable water resulted in unacceptable annual risk without large dilutions or pathogen inactivation. We predicted that 1 user out of 10,000 could be exposed to a 5-day contamination event of undiluted wastewater in the reclaimed, non-potable water system each year to meet the annual benchmark risk of 10^−4^ infections per person per year; whereas, 1 user out of 1000 could be exposed to a 5-day contamination event of undiluted reclaimed water in the potable water each year. Overall, the predicted annual risks support the use of previously derived non-potable reuse treatment requirements for a variety of ONWS sizes and support the prioritization of protective measures to prevent the intrusion of wastewater into domestic ONWS.

## 1. Introduction

There is growing interest across the United States in onsite non-potable water systems (ONWS) to sustainably manage water [1]. These systems collect various sources of water (e.g., greywater and wastewater) and treat it locally for both indoor and outdoor uses (e.g., toilet flushing and irrigation). Example domestic systems include the Solaire residential building in New York City and the Pimpama–Coomera residential district in Australia. The health risk from exposure to enteric pathogens from designated uses of ONWS following risk-based treatment requirements (e.g., [2]) is considered acceptable (i.e., less than the selected annual benchmark). However, the health impact from unintended exposure events in domestic (or office) ONWS, such as cross-connection contamination events or accidental ingestion, remains uncharacterized in scientific literature, with the exception of one well-studied event in the Netherlands [3]. This work explores the possible health impact of cross-connections in domestic (or office) ONWS, defined here as intrusion of lesser quality water into a water system, based on reported events from the literature as well as using quantitative microbial risk assessment (QMRA) [4].

QMRA is a scientific approach to estimate the potential human health risks resulting from exposures to microbial hazards (i.e., human pathogenic viruses, protozoa, and bacteria) [4] and has been used to characterize the risk from intrusions of wastewater into potable water systems [5–7] as well as accidental ingestions of poorly treated greywater (summarized in a review by Schoen and Garland [8]). ONWS are novel in that they present the opportunity for two unique contamination events in domestic (or office) settings. The first is contamination of non-potable, reclaimed water (i.e., treated wastewater or greywater) with wastewater or greywater. The predicted probability of infection from exposure to wastewater (or greywater) is high for a potable volume (as demonstrated by QMRA of contaminated drinking water [5–7]); however, the predicted risk from exposure to a non-potable volume from domestic use, which is extremely small in comparison, is unreported. The second type of event is contamination of potable water with reclaimed water. The risk-based treatment requirements for ONWS, expressed as log reduction targets (LRTs), included a safety factor that provides extra protection against this second type of event [2,9]. However, the safety factor did not account for the full range of possible contamination event conditions in ONWS. The chlorine residual in the water system also acts as an extra protective barrier against bacteria and, under certain conditions, viruses in this event; however, it is less effective against protozoa [10]. The objectives of this work were to estimate the microbial risks associated with a range of possible cross-connection events in ONWS and to assess the sensitivity of domestic, indoor ONWS LRTs to the cross-connection assumptions.

## 2. Materials and Methods

### 2.1. Literature Review

We conducted a review of peer-reviewed literature to identify the characteristics of cross-connection contamination events between potable and reclaimed water (using search terms “drinking water and cross connection”). Databases included Biological Abstracts, Environmental Databases, PubMed, Sci Search, Current Contents, and Waternet. Separately, we searched Google Scholar for characteristics of contamination events of non-potable, reclaimed water (using search terms “non-potable and cross connection or intrusion” and “reclaimed and cross connection or intrusion”). We also informally requested information on events in ONWS from the members of the Blue Ribbon Commission for ONWS [11]. We recorded data on cross-connection locations, event frequencies, size (as percentage of the population served), duration, and contamination magnitude, when each was available.

### 2.2. Pathogen Health Impact

We estimated the pathogen health impact from two cross-connection types. The first was non-potable, reclaimed water (i.e., wastewater or greywater treated to achieve the non-potable LRTs for domestic, indoor uses) contaminated by greywater or wastewater. We refer to this as a “greywater/wastewater to non-potable” event. We define greywater as wastewater from bathtubs, showers, bathroom sinks, and clothes washing machines, excluding toilet, dishwasher, and kitchen sink wastewaters. The second event type was potable water contaminated by non-potable, reclaimed water. We refer to this as a “reclaimed to potable” event. These events were modeled for both reclaimed greywater and reclaimed wastewater, which have different influent pathogen concentrations and LRTs.

The following section describes the QMRA model used to predict both the contamination event probability of infection and the annual probability of infection from year-round, non-potable reuse when including the selected cross-connection event; the model was previously developed to calculate LRTs [9]. Of the relevant human-infectious enteric viruses, bacteria and protozoa [9], we narrowed the list to the dominant reference pathogen in each class (with the exception that both culturable and not readily culturable viruses were included) based on the indoor reuse LRTs (i.e., *Norovirus, Rotavirus, Campylobacter jejuni,* and *Cryptosporidium* spp.). In addition, the QMRA model was used to identify target cross-connection event characteristics, which resulted in an annual probability of infection equivalent to the selected annual health benchmark. In the United States, an infection risk of 10^−4^ per person per year (ppy) for giardiasis has been suggested for surface water treatment requirements producing drinking water [12,13] and was selected for this work.

#### 2.2.1. QMRA

The contamination event probability of infection (Pinf_event_) was calculated as:

(1) $${\text{Pinf}}_{\text{event}}=S*\left(1-\prod_{n}\left[1-DR\left(\frac{V}{DF}{\text{*10}}^{log_{\text{10}}(\text{C})-\;\text{LRT}\;}\right)\right]\right),$$

where

*S* is the fraction of people in the exposed population susceptible to each reference pathogen

*DR*(...) is a dose-response function for the reference pathogen

*DF* is the dilution factor (i.e., the total volume of water in which the intrusion is mixed)

*V* is the volume of water ingested per day

*n* is the number of days of exposure

*C* is the pathogen concentration in the wastewater or greywater

LRT is the total log reduction target for each reference pathogen

The Pinf_event_ in Equation (1) was calculated from a daily pathogen dose accumulated from potable or non-potable ingestion (see Section 2.2.2). During an event, we assumed that all of the water ingested per day was contaminated. A viability factor was not included in Equation (1); hence, we assumed that 100% of the reported protozoa, bacteria, and viruses were viable. The non-potable, indoor LRTs [9] were applied to the raw wastewater or greywater in the reclaimed to potable event, but held at zero for the greywater/wastewater to non-potable event. Equation (1) did not include additional pathogen removal beyond the LRT, such as inactivation from the chlorine residual in the water distribution system.

The pathogen-specific annual probability of infection (Pinf_annual_) from non-potable reuse was estimated as the weighted sum of risk across users:

(2) $$Pinf_{\text{annual}}={\text{fr}}^{*}(1-\left(1-{\text{Pinf}}_{\text{non-potable}}\right)*(1-{\text{Pinf}}_{\text{event}}))+(1-\text{fr})*{\text{Pinf}}_{\text{non-potable}}$$

where

fr is the cross-connection exposure fraction (i.e., exposed users/total users per year)

Pinf_non-potable_ is the annual risk from non-potable reuse without a cross-connection event

Equation (2) was used to solve for the target cross-connection exposure fraction or target dilution, i.e., the fraction of users, or dilution, which resulted in an annual probability of infection equivalent to the selected annual health benchmark (i.e., 10^−4^ infections ppy) for an individual pathogen. Pinf_non-potable_ was estimated using Equation (1) with DF = 1 and *n* = 365.

We treated the highly variable inputs (i.e., pathogen concentration) probabilistically. Monte Carlo analyses were implemented with 10,000 iterations (each with 365 days) in R 3.2.3 [14] to capture the variation in pathogen concentration. The highly uncertain inputs (i.e., the cross-connection characteristics) were treated deterministically and explored by constructing various scenarios based on literature review results (see Section 3.1). The following sections describe the remaining inputs to Equations (1) and (2).

#### 2.2.2. Exposure Routes

For the greywater/wastewater to non-potable scenario, the annual risk accounted for exposures to pathogens from year-round, non-potable reuse as well as the selected contamination event. The ingested volume for non-potable indoor use (i.e., toilet flush water and clothes washing) was 4 × 10^−5^ L of water consumed per day for 365 days a year. This value is uncertain and includes inhalation of aerosols and fomite-hand-mouth exposures (for a full discussion on indoor use ingestion, please refer to [9]). We assumed 100% partitioning and/or recovery for aerosol or fomite-hand-mouth exposures, and thus a partitioning coefficient was not included in Equation (1). During the greywater/wastewater to non-potable contamination event, the ingestion volume remained at 4 × 10^−5^ L of water consumed per day.

For the reclaimed to potable scenario, again, the annual risk accounted for exposures to pathogens from year-round, non-potable reuse as well as the selected event. The exposure assumptions for the non-potable indoor use were the same as described for the first scenario. During the reclaimed to potable contamination event, the potable ingestion volume was set at 2 L, which is the 90th percentile of consumer ingestion across ages [15].

#### 2.2.3. Pathogen Dose-Response

We selected commonly used dose-response models that relate a healthy adult’s dose to a probability of infection based on ingestion (see [9] for more details). For *Rotavirus,* we selected the approximate beta-Poisson model (for doses in focus-forming units (FFU)) by Haas et al. [4]. For *Campylobacter jejuni,* we selected the approximate beta-Poisson model (for doses in colony forming units (CFU)) by Medema, et al. [16]. For the remaining pathogens, there are multiple dose-response models, with different mechanistic assumptions and dose-response data. For *Norovirus* (doses in genome copies (gc)), we selected two models that capture the range of risk predicted across models. The hypergeometric model for disaggregated viruses developed by Teunis, et al. [17] was selected as the upper bound, which is generally used in QMRA and predicts relatively high risks among the available models in the relevant dose range. We selected the fractional Poisson model proposed by Messner, et al. [18] for the lower bound; it predicts similar risks as the majority of the published *Norovirus* dose-response models with good empirical fit to the available data [19]. For *Cryptosporidium* spp. (doses in oocysts), we adopted the fractional Poisson model proposed by Messner and Berger [20] for the upper bound and the an exponential model from the U.S. EPA Long Term 2 Enhanced Surface Water Treatment Rule (LT2) Economic Analysis with r = 0.09 [21] for the lower bound. However, the dose-response models do not include data for the low-dose exposures, and the true dose-response relationship at these levels of exposure remains uncertain.

We adopted the conservative assumption that 100% of the population of healthy adults is potentially susceptible to all pathogens (i.e., *S* = 1 in Equation (1) ), with one exception. For *Rotavirus,* we used a dose-response model for healthy adults but assumed that only young children were susceptible (6% of the population, which is likely high given widespread vaccination) [22]. Note that the fractional Poisson model partitions the exposed population into fully susceptible and fully non-susceptible fractions [18].

#### 2.2.4. Characterization of Pathogens in Waters

In the absence of a distributed greywater or wastewater study with sufficient pathogen monitoring data described in literature, we adopted previously simulated pathogen concentrations from an epidemiology-based model [23]. The epidemiological approach combined data describing population illness rates (as a surrogate for infection) and pathogen shedding characteristics (fecal concentration and shedding duration) with levels of fecal contamination in source waters (based on indicator bacteria) to model the occurrence and concentrations of pathogens in those waters. Three different collection system sizes and types were modeled separately: 1000-person residential collection, 40,000-person residential collection, and office building (4500-person) collection, as previously described [24]. These were selected to explore the impact of collection size and water quality type. Collection size (contributing population) is an important driver of pathogen densities in decentralized systems due to the sporadic nature of pathogen occurrences among small populations; as collection size increases, pathogens occur more frequently in wastewaters yet are diluted by an increased flow from additional non-infected individuals [23]. The Supplementary Material presents the pathogen concentrations in the untreated wastewater and greywater for each of the three collection system sizes (Tables S1 and S2).

#### 2.2.5. Pathogen LRTs

Instead of adopting the treatment performance of a selected treatment technology, we adopted previously-reported indoor, non-potable LRTs (at the 1000-person scale) for simulating the reclaimed water quality [9]. We did this to make the results generalizable to any treatment train that achieves the LRTs.

### 2.3. Non-Potable LRTs without Cross-Connection Event

Using the same data and methods previously used to calculate the LRTs for ONWS [9], which included a safety factor of one reclaimed to potable cross-connection event affecting 10% of the population one day per year, we simulated the LRTs for each reference pathogen without the cross-connection event to see the influence of the previous cross-connection assumptions on the LRTs.

## 3. Results

### 3.1. Cross-Connection Characteristics

Our literature review for cross-connection characteristics returned no results for ONWS events in the United States. In addition, we found no published data or reports from the members of the Blue Ribbon Commission. We did receive anecdotal evidence, but without accompanying data. The lack of data corroborates previous findings, “Despite the significant presence of dual reticulation systems cross the USA, very limited research into cross-connection events and cross-connection detection has been published.” [25]

Cross-connection event characteristics in ONWS, such as event duration, exposure fraction, and yearly event rate, were reported in peer-reviewed literature from Australia and the Netherlands [3,25–29]. We used this literature to define a possible range of each characteristic to bound our QMRA input parameters. Only reclaimed to potable events were reported; these findings were assumed to hold for the wastewater/greywater to non-potable events.

Data on contamination magnitude was not reported (i.e., pathogen concentration); so, we evaluated a wide range of possible intrusion dilutions (1-part intrusion water to 1–1000 parts total water). The exposure fraction was reported as the fraction of connections (or people served) per year. At the low end, Australian Recycled Water Guidelines recommend an annual cross-connection frequency of around 1 event in 1000 dwellings per year [29]. At the high end, 630 of 4400 dwellings in Pimpama Coomera, Australia were supplied with contaminated drinking water at the initial startup for a period of 4 days [30]. We modeled the range in exposure fraction from 0.001 to 1. We adopted the reported event duration range, from 1 to 30 days [3,25–29].

### 3.2. Daily Event Risk

#### 3.2.1. Reclaimed to Potable Water Event

The pathogen-specific, 95th percentile event probability of infection for ingestion of undiluted domestic, non-potable reclaimed wastewater (from a 1000-person collection) at a potable volume was 8.4 × 10^−4^ per person per event (ppe) and 6.7 × 10^−4^ ppe for reclaimed greywater (complete results in Supplementary Material Figures S1–S6). Generally, the two pathways shared similar risks across pathogens because the pathogen-specific LRTs applied to the raw wastewater or greywater equalized the risk from each pathogen in the reclaimed water to the selected benchmark. The 95th percentile risk decreased by 1 log10 as dilution increased by 1 log10. The median daily risks were zero for all pathogens except *Norovirus* (e.g., ~10^−5^ infections ppe for undiluted reclaimed wastewater) due to the low pathogen occurrence in the collected waters (see Tables S1 and S2). The risk was greater than zero for *Rotavirus, Cryptosporidium* spp., and *Campylobacter* at the 81st, 87th, and 73rd percentiles, respectively (e.g., 2.8 × 10^−5^, 2 × 10^−7^,6.1 × 10^−10^ infections ppe for undiluted reclaimed wastewater).

The daily risks for the 40,000-person collection systems (Figures S1 and S4 for reclaimed wastewater and greywater, respectively) were slightly greater than the 1000-person system (Figures S2 and S5) because the LRTs were designed for the 1000-person system, whereas the frequency of pathogen occurrences increased with contributing population [23,24]. The predicted event risk from the reclaimed office wastewater contamination event (Figure S3) was the same order of magnitude as the predicted event risk of domestic reclaimed wastewater from a 40,000-person system. Whereas, a contamination event with reclaimed office greywater (Figure S6) resulted in a predicted event risk similar to the 1000-persondomestic reclaimed greywater contamination event for all pathogens but *Norovirus* (Figure S6), which was lower due to the higher microbial quality of bathroom sink *g*reywater collected from offices vs. combined greywater (i.e., including showers and laundry) collected from domestic residences [23,24].

#### 3.2.2. Greywater/Wastewater to Non-Potable

**Figure 1.**
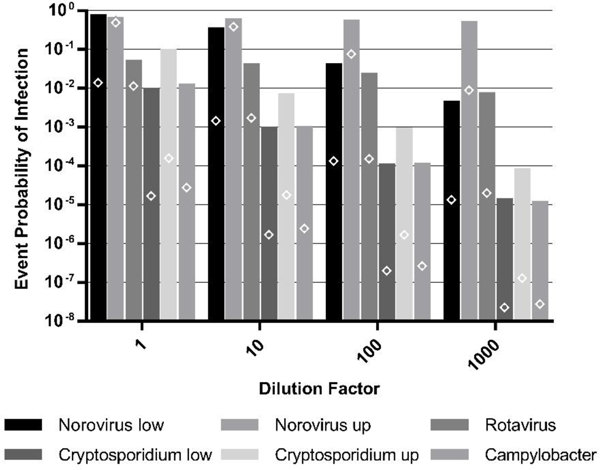
The 95th percentile event probability of infection for ingestion of domestic, non-potable reclaimed wastewater contaminated by domestic wastewater (bars) or domestic, non-potable reclaimed greywater contaminated by domestic greywater (diamonds), both generated by a 1000-person residential collection system. The dilution factor is expressed as 1-part intrusion water: X parts total water. Both upper- (up) and lower- (low) bound dose-response presented.

The pathogen-specific 95th percentile event probabilities of infection for ingestion of domestic, non-potable water contaminated by domestic wastewater or greywater Eire presented in Figure 1 for a range of possible intrusion dilutions. The pathogens head unique predicted risks biased in the pathogen densities in the intrusion water. The results in 1 were generated using pathogen densities from the 1000-person collection system. The Supplementary Material Figures S13–S18 present the other greywater and wastewater contamination events. The same trends across the three different collection system sizes and types were present as described in Section 3.2.1. Generally, *Norovirus* was the reference pathogen with the greatest event risk, noting that chlorine residual disinfection was not included in the model. Notice that the difference in the *Norovirus* risks for wastewater increased as the dilution increased. This is due to the *Norovirus* dose-response relationships, which predict similar risks at higher doses, but diverge at low doses.

Similar to the resulSs in Section 3.2.1, the medisn risks (not shown) were zero for gill pathogens except *Norovirus* (e.g., 8 × 10^−2^−5 × 10^−1^ infections ppe for undilutedwastewater and 2 × 10^−4^ - 1 × 10^−1^ infections ppe for undiluted greywater from 1000-person collection systems) due to the low pathogen occurrence. The risk exceeded zero fot *Rotavirus, Cryptosporidium* spp., and *Campylobacter* at the; 80th, 89th, and 73rd percentiles, respectively (e.g., 1 × 10^−4^, 6 × 10^−6^-2.0 × 10^−5^, 7 × 10^−9^ infections ppe for undiluted wastewater and 2 × 10^−5^, *2* × 10^−7^−2 × 10^−6^, 7 × 10^−10^ infections ppe for undiluted greywater from 1000-person collection systems).

#### 3.2.3. Comparison of Intrusion Scenario Daily Event Risks

We compared the event risks (assuming one day of ingestion and no dilution) across intrusion scenarios using the dominant pathogen from each scenario. The intrusion of wastewater into non-potable water had the highest event risks (i.e., 95th percentile of *Norovirus* was >0.60 infections ppe across collection size types). Next highest was the intrusion of greywater into non-potable water (i.e., 95th percentile of *Norovirus* for the 1000-person collection was 1.4 × 10^−2^ and 0.49 infections ppe for the lower and upper-bound dose-response models). Reclaimed wastewater and domestic greywater to potable followed (i.e., 95th percentile of 8.4 × 10^−4^ jape; and 6.7 × 10^−4^ ppe across pathogens for 1000-person collection). The lowest event risk was from intrusions of reclaimed office greywater into potable water i.e., 95th percentile of 2.5 × 10^−4^ infections ppe across pa(hogens).

When focusing on *Cryptosporidium* spp. risk (using the upper bound dose-response), since it is not affected by chlorine residual disinfection, the predicted event risks for cross-connections between wastewaters and non-potable water were reduced compared to the dominant pathogen. The intrusion of wastewater into non-potable water still had the highest event risks (i.e., 95th percentile > 9.1 × 10^−2^ infections ppe across the three different collection system sizes). The intrusion of greywaters into non-potable water had events risks (i.e., 95th percentile of 1.5 × 10^−4^ to 2.2 × 10^−3^ infections ppe across the three different collection system sizes) which were similar in range to the reclaimed wastewater and greywater to potable (i.e., 95th percentile of 2.5 × 10^−4^ to 6.2 × 10^−3^ infections ppe across the three different collection system sizes and water types).

### 3.3. Predicted Annual Risk

#### 3.3.1. Reclaimed to Potable

**Figure 2.**
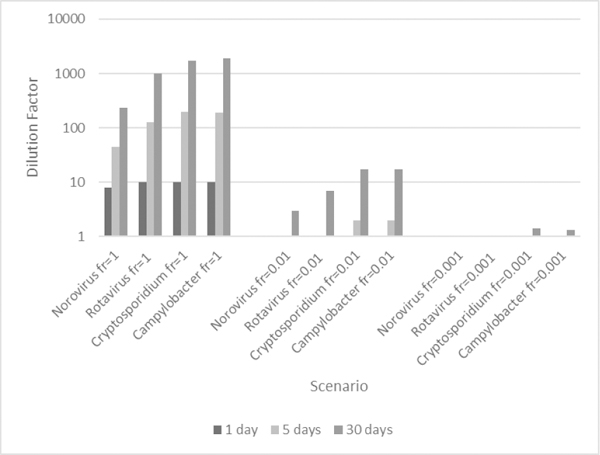
Target dilution factor foo intrusion of non-potable reclaimed wastewater (generated by 1000-person collection and treated for indoor reuse) to potable water that results in 95th percentile annual risk of infection from non-potable domestic, indoor reuse equivalent to the benchmark (10^−^4 ppy). The intrusion event duration was either 1, 5, or 30 days for various fractions of the population exposed to the intrusion event (fr). The dilution factor is expressed as 1-part intrusion water: X parts total water.

The event risks (presented in Section 3.2) only accounted for exposures to pathogens from an intrusion event. In contrast, the predicted annual risk estimated the risk from year-round, non-potable reuse for users plus a contamination event of selected duration and dilution for a fraction of the users. Since the actual dilution of these events remains unknown, the pathogen-specific target dilution factor of reclaimed non-potable water (i.e., that results in a 95th percentile annual probability of infection of 10^−4^ ppy) wan estimated for a range of possible exposure fractions and durations (Figure 2). The results in Figure 2 were generated using wastewater pathogen densities from the 1000-person collection system. The Supplementary Material Figures S7–S12 present the other greywater and wastewater contamination results.

The pathogens (and upper and lower dose-response relationships, when applicable) shared similar dilutions for a duration of one day because the pathogen-specific LRTs applied to the raw wastewater targeted the same acceptable risk. However, the pathogen-specific dilutions varied with longer event durations since more concentrations were sampled from the influent distributions, and *Cryptosporidmm* spp. and *Campylobacter* generally dominated.

For 1-day events with a small exposure fraction (i.e., 0.01 or less) or e-vents with an exposure fraction of 0.001 or less (for e-vent durations of 30-days or less), the target dilution factors for the contamination were approximately 1 for all water types and population sizes, meaning that the systems were protected by the LRTs (derived by the 1000-person system). The target dilution factors were greater than 1 when the event duration increased to 5-days for exposure fractions of 0.01 or more for all water types and population sizes. Therefore, some dilution (or additional inactivation) of the contamination was required to meet the annual health benchmark.

#### 3.3.2. Wastewater/Greywater to Non-Potable

**Figure 3.**
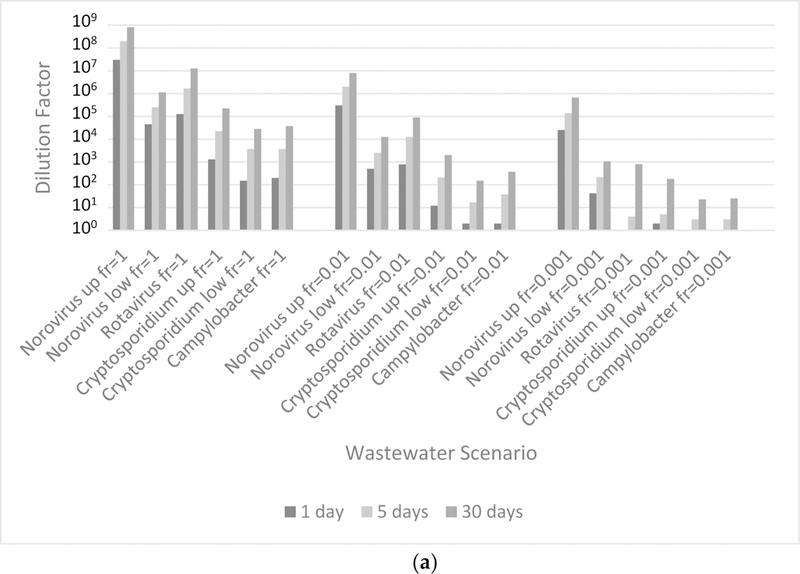

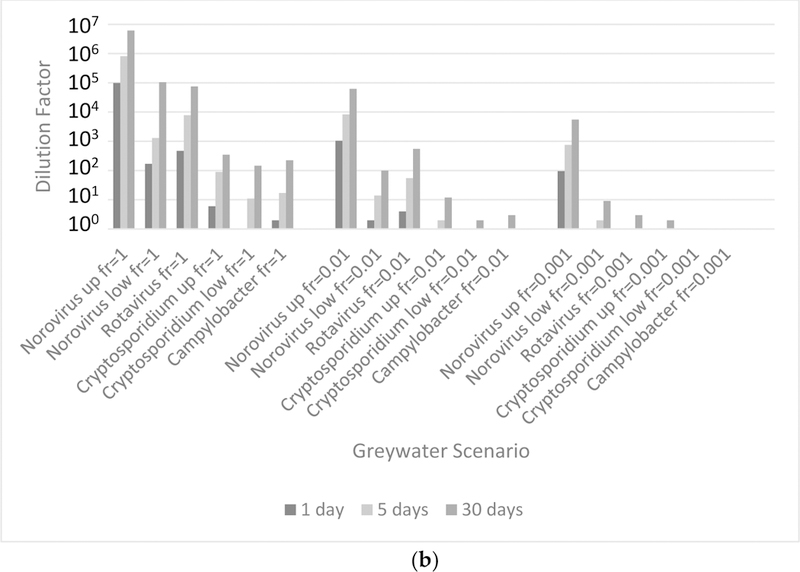
Target dilution factor for intrusion of (a) wastewater and (b) greywater (generated by 1000-person collection) to non-potable water that results in a 95th percentile annual risk of infection from non-potable domestic, indoor reuse equivalent to the benchmark (10^−4^ ppy). The intrusion event duration was either 1, 5, or 30 days for two alternative fractions of the population exposed to the intrusion event (fr = 1, 0.01, or 0.001). The dilution factor is expressed as 1-part intrusion water: X parts total water. Both upper- (up) and lower- (low) bound dose-response presented.

The target dilutions presented in Figure 3 for intrusions of wastewater and greywater into non-potable water were generated using pathogen densities from the 1000-person collection system. The Supplementary Material Figures S19–S24 present the other greywater and wastewater contamination results. Generally, the viral reference pathogens required the greatest target dilution factor (again, disinfection from the chlorine residual was not considered). Since the target dilution factor was greater than 1 across pathogens for the selected scenarios, dilution (or additional inactivation) of the contamination was required to meet the annual health benchmark.

#### 3.3.3. Comparison of Target Dilution Factor

The target dilution factors for the wastewater/greywater to non-potable event were much greater than those required for the reclaimed to potable event. For example, a 1-day intrusion of wastewater (or greywater) into the non-potable, reclaimed water for an exposure fraction of 0.001 had a target dilution factor exceeding 10,000 (or 95 for greywater) for *Norovirus* using the upper-bound dose-response; 42 (or 1 for greywater) for *Norovirus* using the lower-bound dose-response; and approximately 1 for *Cryptosporidium* spp. using the upper-bound dose-response. Whereas, under the same event conditions, the reclaimed to potable event required no additional dilution.

#### 3.3.4. Comparison of Target Exposure Fraction

We calculated the target exposure fraction for which a system with uncharacterized dilution (conservatively assumed to be 1) and <5-day duration should aim to meet the health benchmark of 10^−4^ infections ppy. We compared the target fractions across intrusion scenarios using the dominant pathogen from each scenario as well as *Cryptosporidium* spp. using the upper bound dose response. We converted the target fractions into ratios of affected to total connected population and generalizrd results across the three sized systems when possible. The wastewater to non-potable event had the lowest target fraction (i.e., 1:10,000 per year based on *Norovirus* with the upper- or lower-bound dose-response or *Cryptosporidium* spp.). Reclaimed wastewater and domestic greywater to potable followed (i.e., roughly 1:1000 per year across pathogens). The highest target fraction was from intrusions of reclaimed office greywater into potable water (i.e., 1:100 per year across pathogens).

The target fraction for intrusion of domestic greywaters into non-potable water varied, depending on the pathogen and the selected dose-response (i.e., roughly 1:10,000 per year based on *Norovirus* with the upper bound dose-response; 1:1000 per year based on *Rotavirus* and *Norovirus* with the lower bound dose-response; and 1:100 for *Cryptosporidium* spp.). Similarly, the target fraction for intrusions of office greywater into non-potable varied (i.e., roughly 1:8000 per year based on *Norovirus* with the upper bound dose-response; 1:50 for *Norovirus* with the lower bound dose-response; and 1:10 per year for *Cryptosporidium* spp.).

### 3.4. LRTs without Cross-Connection

The LRTs for non-potable domestic indoor use (for toilet flushing and clothes washing) given the 10^−4^ ppy (infection) benchmark were recalculated assuming no exposures to accidental ingestion or unprotected cross-connections. The results are presented in Table 1 for each reference pathogen alongside the original LRTs used in (Equation 1) (which included additional protection against cross-connection events) [2,9]. The LRTs decreased by roughly 1 logi_0_ or less, except for the *Norovirus* LRT which decreased by 1.4 log_10_.

## 4. Discussion

### 4.1. How Important Are Cross-Connection Events between Potable and Non-Potable Water?

The intrusion event QMRA results (Section 3.2) corroborate the available (but limited) literature, which suggests that the microbial health impact of ingesting potable water contaminated by reclaimed water varies [25]. For domestic ONWS treated according to risk-based LRTs, the increase in annual health risk from contamination of potable water is negligible if the fraction exposed is low, with some dilution, and with a duration of 5 days or less (see Section 3.3 for target dilution values). However, as the exposure fraction and duration increase, the cross-connection risk becomes much greater than the relatively small, acceptable risk from domestic, non-potable use of reclaimed water. These longer events or events that effect a large percentage of the population will push the system out of compliance, even if the chlorine residual is maintained, given the lack of sensitivity of protozoa to chlorine disinfection. Note that since domestic ONWS may be small (i.e., 1000 persons or less) a system may exceed the health benchmark (i.e., >10^−4^ infections ppy) because of an intrusion event and have no reported illnesses.

### 4.2. How Important Were the Cross-Connection Assumptions Built into the Domestic LRTs?

The cross-connection exposure fraction and dilution assumptions built into the domestic ONWS LRTs act as a safety factor to provide some extra protection from these types of events. The extra protection requires about 1 log10 increase in pathogen reduction for indoor, non-potable uses (see Section 3.4), which leaves the bulk of the LRT for protection from intended uses. The “right” amount of extra protection provided by the safety factor remains debatable. This work demonstrates that the current safety factor is effective for short-term, low magnitude reclaimed to potable cross-connection events in ONWS. The extra protection seems reasonable, even if events remain largely unreported, given the inherent possibility of unintended exposures to indoor, non-potable water. If contamination events in ONWS occur more frequently and widespread, then other protective measures are likely a better solution than increasing the LRT. Similarly, the LRTs did not include a safety factor for wastewater/greywater to non-potable contamination events, which are better managed in the distribution system or on premise.

### 4.3. What Type of Contamination Events Have the Most Predicted Health Impact in ONWS?

For domestic ONWS, the predicted event microbial health risk from exposure to untreated wastewater in non-potable water was very high, even at high dilutions, despite the small volume consumed. Potable contamination events were outside the scope of this analysis but would result in a higher predicted event risk than non-potable given the larger volume consumed (holding assumptions about dilution and duration constant). It is difficult to determine the annual health burden of any of the selected event types given the lack of data on event characteristics in ONWS.

### 4.4. What Is the Acceptable Fraction of ONWS Users Impacted by Contamination Events?

The fraction of ONWS users who can be impacted by a contamination event, and still meet the annual health benchmark, varies depending on the type and characteristics of the occurring cross-connections. We calculated conservative targets assuming no dilution of the contaminant and a 5-day event duration. The target exposure fraction was roughly 1 impacted user per 1000 users for cross-connection events between non-potable, reclaimed and potable water as well as between greywater and non-potable, reclaimed water. This corroborates the Australian Recycled Water Guidelines annual cross-connection frequency of around 1 event in 1000 dwellings per year [29]. The target exposure fraction for contamination of non-potable water by wastewater was 1 impacted user per 10,000 users. Again, these fractions assume no dilution of the contamination, and the target exposure fractions would decrease if information on dilution was incorporated.

### 4.5. Important QMRA Assumptions

The dose-response assumptions and limitations were previously discussed with the most obvious being the lack of dose-response data at low doses [9]. As described in Section 2.2.3, there are numerous dose-response relationships for *Norovirus*. The selected dose-response relationships diverge at low doses, and as a result, the predicted target dilutions for contamination of reclaimed water by untreated wastewater differ by three orders of magnitude (Figure 3). At higher doses, like those from ingestion of undiluted wastewater, the relationships predict similar risk (Figure 2).

*Norovirus* presents a unique case since the commonly used molecular-based approach cannot differentiate infectious or inactivated viruses. Therefore, the fraction of total to infectious genomes in the dose-response studies was unknown. This is a limitation when we characterize the *Norovirus* concentration in collected or treated wastewater and greywater since the fraction of total to infectious genomes likely varies across environments, but we assumed that the fraction was the same as in the human challenge study. This limitation is important for intrusions of untreated wastewater and greywater, in which viruses dominated the predicted health impact. This is less of a concern for the contamination of potable water with reclaimed water since the risks across pathogens is similar.

For the potable water contamination events, we assumed 2 L of contaminated water were ingested per person per day given that the duration was 1 day or more. If the event duration was shorter than 1-day, cursory sensitivity analysis of the annual risk to the volume ingested showed a very small change (less than 0.5 log10) when the volume was decreased to 1 L. There is more uncertainty in the non-potable volume ingested. We selected what we believe to be a conservative volume of ingestion for domestic, indoor non-potable use. If the non-potable exposures were smaller, then the contamination event risk would decrease; however, the cross-connection of untreated to non-potable waters is likely to remain a problem given the high pathogen loads in raw wastewaters.

### 4.6. How Can These Findings Be Used?

Foremost, these findings support the prioritization of protective measures to prevent the intrusion of wastewater into domestic ONWS. Second, these findings highlight the potential risk posed by protozoa in contamination events of potable water with non-potable reclaimed water, rather than viruses, which typically dominate the predicted health impact in contamination events with raw wastewaters. Third, the predicted annual risks support the use of the previously derived non-potable LRTs [9] for protection against short-term, low magnitude reclaimed to potable cross-connection events in ONWS as well as protection for larger non-potable systems. In addition, the target exposure fractions can be used as a rough guideline if states require the reporting of contamination events or testing for unprotected cross-connections in ONWS. Finally, the analysis points to the need for more information on the fraction of users that are exposed to cross-connection events and the magnitude of contamination. These data would enable assessment of actual health burdens and the evaluation of whether additional preventative measures are necessary to protect the consumers.

## 5. Conclusions

- There is a lack of data in the United States on cross-connection events in domestic, non-potable water systems
- The predicted health impact of contamination of potable water by reclaimed water varied from negligible to exceeding the annual health benchmark, depending on characteristics of the contamination event.
- Contamination of non-potable reclaimed water with wastewater had higher predicted health impact per event than contamination of potable water by reclaimed water. However, the fraction of users that are exposed and magnitude of these events remain uncertain.
- Domestic non-potable reuse LRTs generally decreased by an order of magnitude (or less) when potable water contamination events were assumed not to exist.
- Based on the QMRA results of cross-connection events, the fraction of users that can be exposed in domestic, onsite non-potable water systems to maintain the annual health benchmark should be less than 1 impacted person per 10,000 users for contamination of non-potable reclaimed water by undiluted wastewater or 1 per 1000 for contamination of potable water by reclaimed wastewaters and domestic greywaters.

## Supplementary Material

Supp Info

## Figures and Tables

**Table 1.** Non-potable indoor use log10 pathogen reductions targets for healthy adults given the 10^−4^ ppy (infection) benchmark for domestic wastewater and greywater assuming either the occurrence of a cross-connection event (CC) [2,9] or no event ^a^.

	*Norovirus* (Genome Copies) ^b^	*Rotavirus* (FFU)	*Cryptosporidium* (oocysts) ^b^	*Giardia* (Cysts)	*Campylobacter* (CFU)	*Salmonella* (CFU)

*Wastewater 1000-person collection*						
Indoor use with CC^a^	11.2/8.4	8.8	6.8/5.9	6.1	6.0	3.8
Indoor use without CC	9.8/7	8.1	6.3/5.4	5.0	5.5	2.9
Greywater *1000-person collection*						
Indoor use with CC ^a^	8.8/6.0	6.4	4.5/3.6	3.8	3.7	1.6
Indoor use without CC	7.9/5.1	6.0	4.1/3.2	3.4	3.3	0.7

a. Assumed 4 × 10^−5^ L of water consumed per day for 365 days a year with 10% of the population ingesting 2 L per day for 1 day of the year [9].

b. Upper and lower bound dose-response presented for *Norovirus* [17,18] and *Cryptosporidium* spp. [20,21].
