# Supplementary material for: Human Health Impact of Cross-Connections in Non-Potable Reuse Systems
Source: Water (Basel). Author manuscript; Available in PMC 2019 Oct 1. (PMC6621552; doi:10.3390/w10101352)
Supplement: Supp Info [file NIHMS1016818-supplement-Supp_Info.pdf]

Human Health Impact of Cross-Connections in Non-potable Reuse Systems

Mary E. Schoen,[a] Michael A. Jahne,[b] and Jay Garland[b]

a. Soller Environmental, Inc., 3022 King St., Berkeley, CA 94703, USA;

mschoen@sollerenvironmental.com

b. U.S. Environmental Protection Agency, 26 W. Martin Luther King Drive, Cincinnati OH 45268,

USA; Jahne.Michael@epa.gov, Garland.jay@epa.gov

Contents

Table S1. Simulated pathogen concentrations [$\log_{10}$ per L] in greywater: percentiles of concentration[a] ... 4

Table S2. Simulated pathogen concentrations [$\log_{10}$ per L] in onsite wastewater: percentiles of concentration[a] ... 4

Figure S1. 95th percentile event probability of infection for ingestion of potable water contaminated by domestic, non-potable reclaimed wastewater from residential collection of 40,000 people. ... 5

Figure S2. 95th percentile e vent probability of infection for ingestion of potable water contaminated by domestic, non-potable reclaimed wastewater from residential collection of 1,000 people.[1] ... 5

Figure S3. 95th percentile event probability of infection for ingestion of potable water contaminated by domestic, non-potable reclaimed wastewater from office collection of 4,500 people. ... 6

Figure S4. 95th percentile event probability of infection for ingestion of potable water contaminated by domestic, non-potable reclaimed greywater from residential collection of 40,000 people. ... 6

Figure S5. 95th percentile event probability of infection for ingestion of potable water contaminated by domestic, non-potable reclaimed greywater from residential collection of 1,000 people. ... 7

Figure S6. 95th percentile event probability of infection for ingestion of potable water contaminated by domestic, non-potable reclaimed greywater from office collection of 4,500 people. ... 7

Figure S7. Safe dilution factor of intrusion of non-potable reclaimed wastewater (generated by 40,000-person residential collection and treated for indoor reuse) to potable water supply that results in a 95th percentile annual risk of infection from non-potable indoor reuse equivalent to 10^{-4} ppy. ... 8

Figure S8. Safe dilution factor of intrusion of non-potable reclaimed wastewater (generated by 1,000-person residential collection and treated for indoor reuse) to potable water supply that results in a 95th percentile annual risk of infection from non-potable indoor reuse equivalent to 10^{-4} ppy. ... 8

Figure S9. Safe dilution factor of intrusion of non-potable reclaimed wastewater (generated by 4,500-person office collection and treated for indoor reuse) to potable water supply that results in a 95th percentile annual risk of infection from non-potable indoor reuse equivalent to 10^{-4} ppy. 9

Figure S10. Safe dilution factor of intrusion of non-potable reclaimed greywater (generated by 40,000-person residential collection and treated for indoor reuse) to potable water supply that results in a....... 9

Figure S11. Safe dilution factor of intrusion of non-potable reclaimed greywater (generated by 1,000-person residential collection and treated for indoor reuse) to potable water supply that results in a 95th percentile annual risk of infection from non-potable indoor reuse equivalent to 10^{-4} ppy. [2] 10

Figure S12. Safe dilution factor of intrusion of non-potable reclaimed greywater (generated by 4,500-person office collection and treated for indoor reuse) to potable water supply that results in a 95th percentile annual risk of infection from non-potable indoor reuse equivalent to 10^{-4} ppy. [2] 10

Figure S13. 95th percentile event probability of infection for ingestion of domestic, non-potable reclaimed wastewater contaminated by domestic wastewater generated by a 40,000-person residential collection system.[1] 11

Figure S14. 95th percentile event probability of infection for ingestion of domestic, non-potable reclaimed wastewater contaminated by domestic wastewater generated by a 1,000-person residential collection system.[1] 11

Figure S15. 95th percentile event probability of infection for ingestion of domestic, non-potable reclaimed wastewater contaminated by domestic wastewater generated by a 4,500-person office collection system.[1] 12

Figure S16. 95th percentile event probability of infection for ingestion of domestic, non-potable reclaimed greywater contaminated by domestic greywater generated by a 40,000-person residential collection system.[1] 12

Figure S17. 95th percentile event probability of infection for ingestion of domestic, non-potable reclaimed greywater contaminated by domestic greywater generated by a 1,000-person residential collection system.[1] 13

Figure S18. 95th percentile event probability of infection for ingestion of domestic, non-potable reclaimed greywater contaminated by domestic greywater generated by a 4,500-person office collection system... 13

Figure S19. Safe dilution factor of intrusion of residential wastewater (generated by 40,000-person collection) to non-potable reclaimed water supply that results in a 95th percentile annual risk of infection from non-potable domestic, indoor reuse equivalent to the benchmark (10^{-4} ppy).[2] 14

Figure S20. Safe dilution factor of intrusion of residential wastewater (generated by 1,000-person collection) to non-potable reclaimed water supply that results in a 95th percentile annual risk of infection from non-potable domestic, indoor reuse equivalent to the benchmark (10^{-4} ppy). [2] 14

Figure S21. Safe dilution factor of intrusion of office wastewater (generated by 4,500-person collection) to non-potable reclaimed water supply that results in a 95th percentile annual risk of infection from non-potable domestic, indoor reuse equivalent to the benchmark (10^{-4} ppy). [2] 15

Figure S22. Safe dilution factor of intrusion of residential greywater (generated by 40,000-person collection) to non-potable water supply that results in a 95th percentile annual risk of infection from non-potable domestic, indoor reuse equivalent to the benchmark (10^{-4} ppy). [2] 15

Figure S23. Safe dilution factor of intrusion of residential greywater (generated by 1,000-person collection) to non-potable water supply that results in a 95th percentile annual risk of infection from non-potable domestic, indoor reuse equivalent to the benchmark (10^{-4} ppy). [2] 16

Figure S24. Safe dilution factor of intrusion of residential greywater (generated by 4,500-person collection) to non-potable water supply that results in a 95th percentile annual risk of infection from non-potable domestic, indoor reuse equivalent to the benchmark (10^{-4} ppy).[2] 16

Table S1. Simulated pathogen concentrations [log_{10} per L] in greywater: percentiles of concentration[a] .

	40000-persons Residential			1000-persons Residential			4500-persons Office		
	**5%**	**50%**	**95%**	**5%**	**50%**	**95%**	**5%**	**50%**	**95%**
Campylobacter	-0.3	1.1	2.7	-Inf	-Inf	1.5	-Inf	-1.2	1.5
Cryptosporidium	-1.3	0.3	1.9	-Inf	-Inf	0.7	-Inf	-Inf	0.7
Norovirus	4.5	5.1	6.0	0.9	3.8	5.7	2.9	3.8	4.7
Rotavirus	2.4	3.6	5.0	-Inf	-Inf	4.1	-Inf	1.5	3.9

a. -Inf indicates a concentration of 0 #/L.

Table S2. Simulated pathogen concentrations [log_{10} per L] in onsite wastewater: percentiles of concentration[a.]

	40000-persons Residential			1000-persons Residential			4500-persons Office		
	**5%**	**50%**	**95%**	**5%**	**50%**	**95%**	**5%**	**50%**	**95%**
Campylobacter	2.4	3.7	5.1	-Inf	-Inf	4.2	-Inf	2.3	5.2
Cryptosporidium	1.4	3.0	4.2	-Inf	-Inf	3.4	-Inf	-Inf	4.4
Norovirus	7.1	7.5	8.1	3.5	6.5	8.2	6.5	7.5	8.6
Rotavirus	5.1	6.2	7.4	-Inf	-Inf	6.8	-Inf	5.0	7.6

a. -Inf indicates a concentration of 0 #/L.

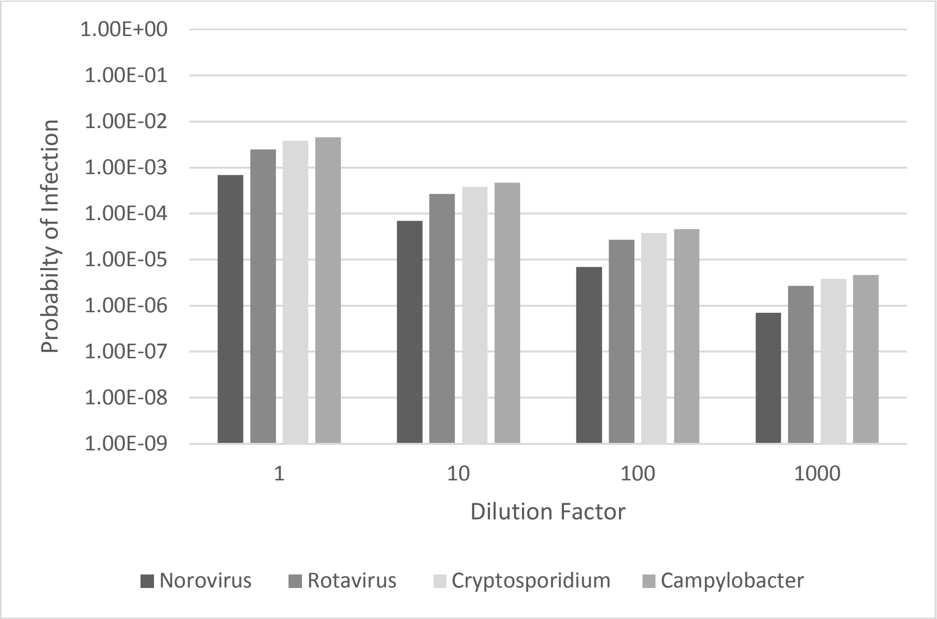

Figure S1. 95th percentile event probability of infection for ingestion of potable water contaminated by domestic, non-potable reclaimed wastewater from residential collection of 40,000 people.[1] .

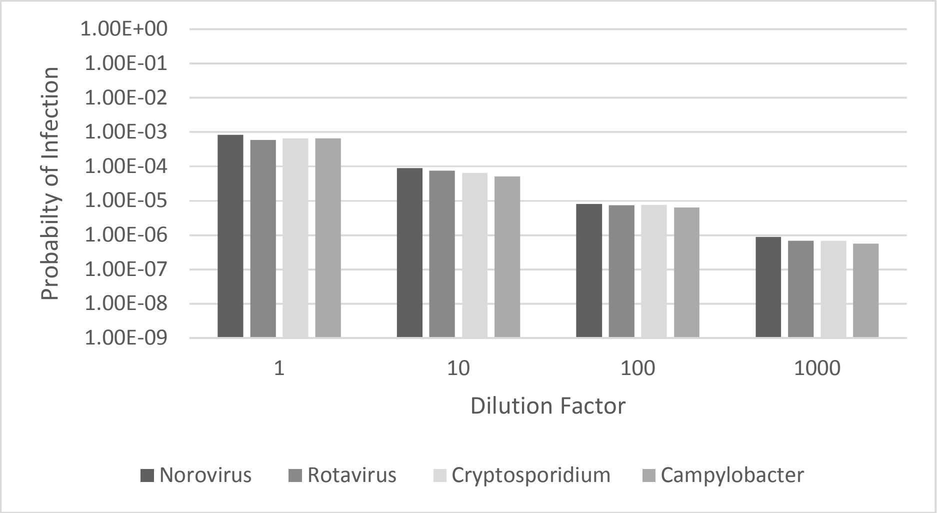

Figure S2. 95th percentile event probability of infection for ingestion of potable water contaminated by domestic, non-potable reclaimed wastewater from residential collection of 1,000 people.[1] .

[1] The dilution factor is expressed as 1 part intrusion water: X parts total water. *Norovirus* lower-bound dose-response results and *Cryptosporidium* upper-bound dose-response results presented.

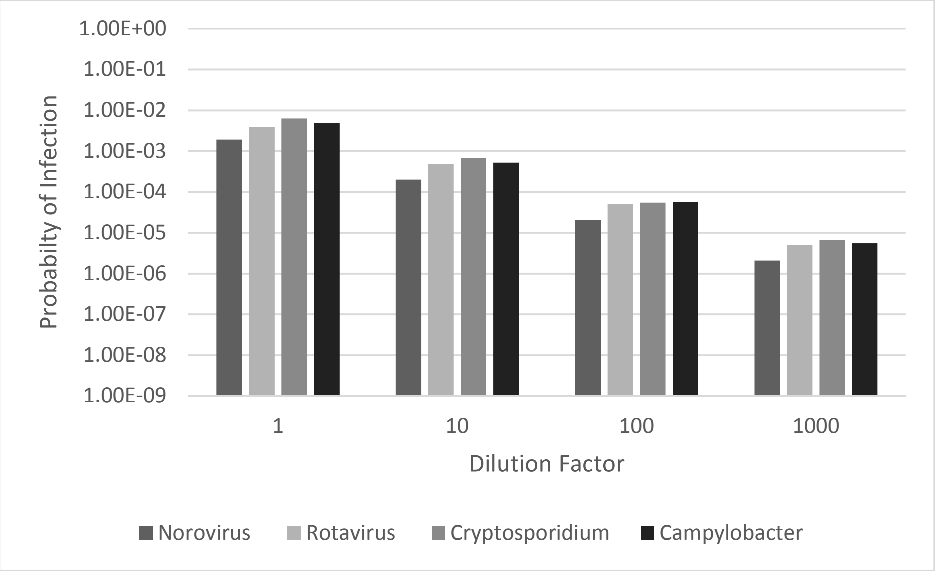

Figure S3. 95th percentile event probability of infection for ingestion of potable water contaminated by domestic, non-potable reclaimed wastewater from office collection of 4,500 people.[1] .

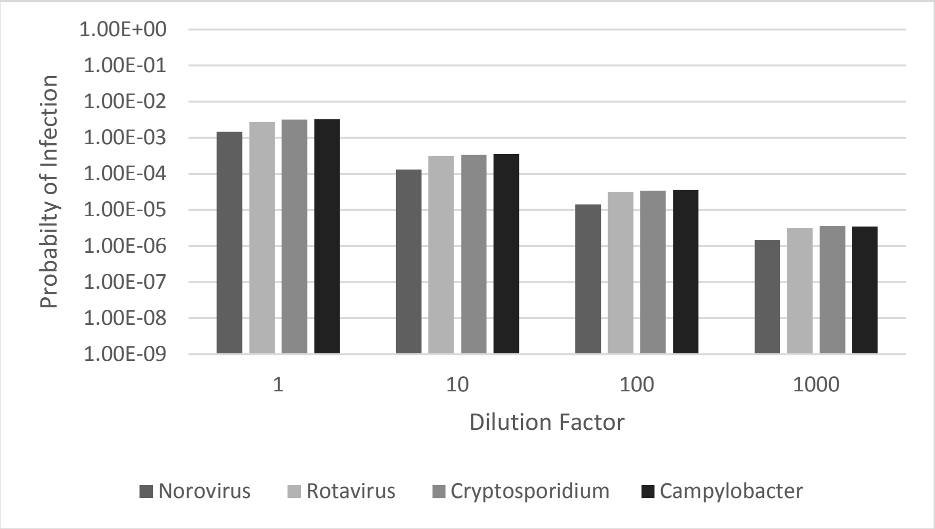

Figure S4. 95th percentile event probability of infection for ingestion of potable water contaminated by domestic, non-potable reclaimed greywater from residential collection of 40,000 people. [1] .

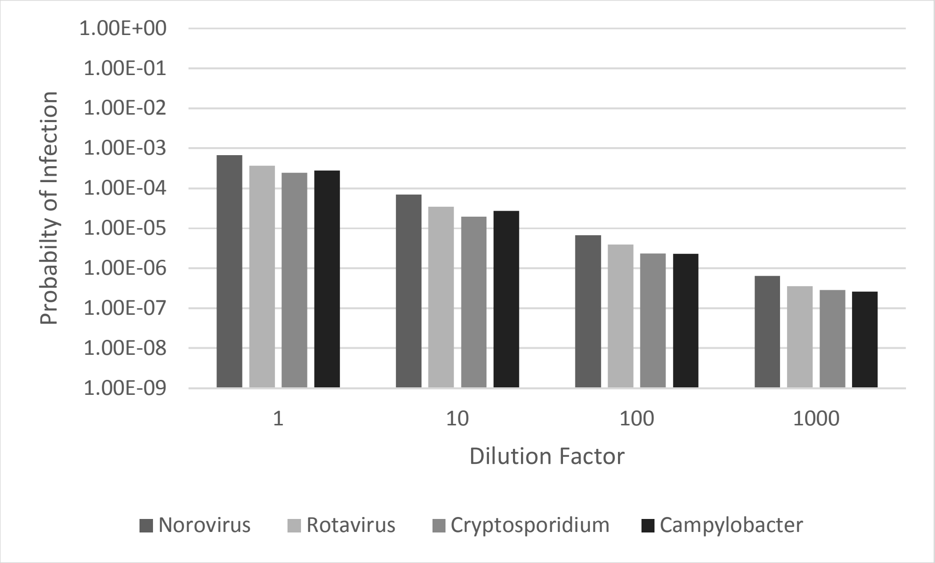

Figure S5. 95th percentile event probability of infection for ingestion of potable water contaminated by domestic, non-potable reclaimed greywater from residential collection of 1,000 people. [1] .

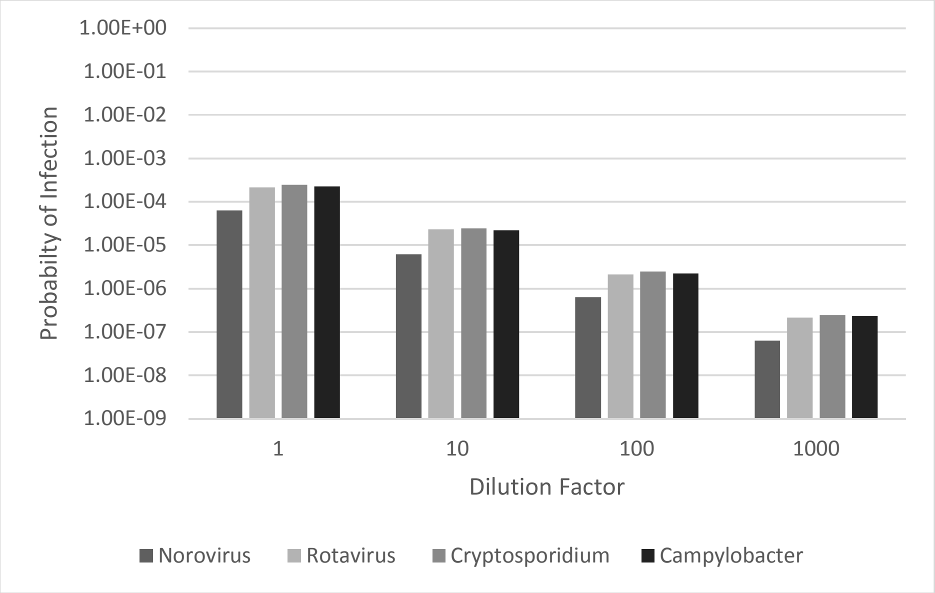

Figure S6. 95th percentile event probability of infection for ingestion of potable water contaminated by domestic, non-potable reclaimed greywater from office collection of 4,500 people. [1] .

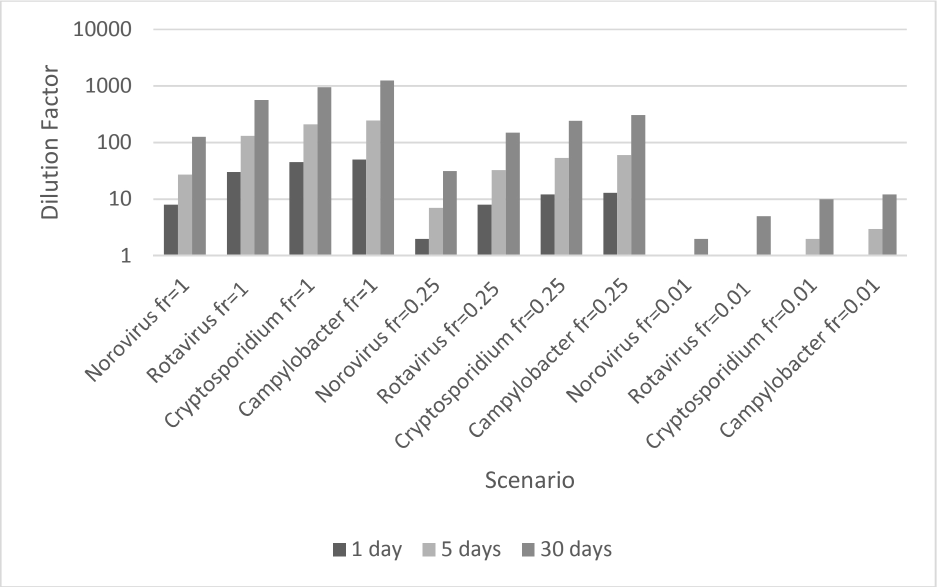

Figure S7. Safe dilution factor of intrusion of non-potable reclaimed wastewater (generated by 40,000-person residential collection and treated for indoor reuse) to potable water supply that results in a 95th percentile annual risk of infection from non-potable indoor reuse equivalent to 10^{-4} ppy.[2].

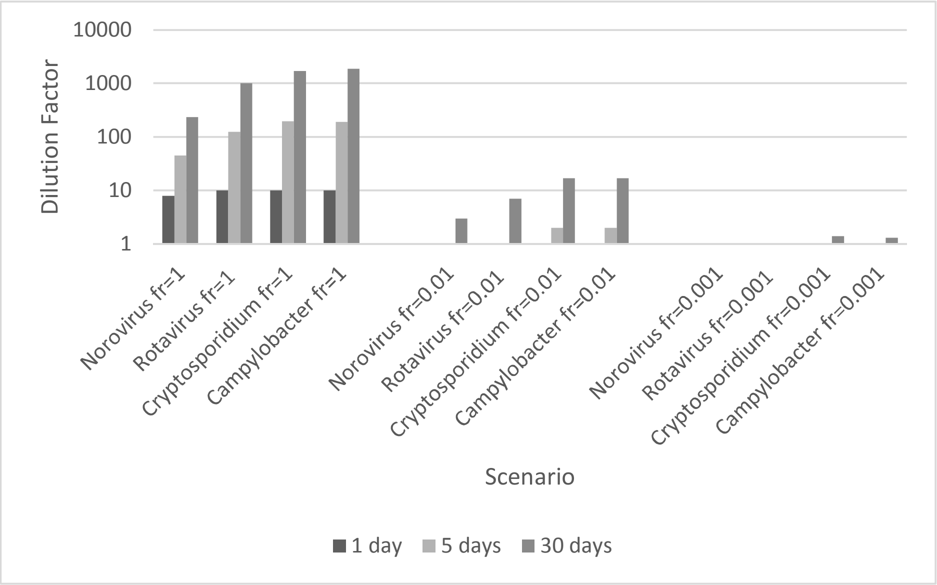

Figure S8. Safe dilution factor of intrusion of non-potable reclaimed wastewater (generated by 1,000-person residential collection and treated for indoor reuse) to potable water supply that results in a 95th percentile annual risk of infection from non-potable indoor reuse equivalent to 10^{-4} ppy. [2].

[2] The intrusion event duration was either 1, 5 or 30 days for various fractions of the population exposed to the intrusion event (fr). *Norovirus* lower-bound dose-response results and *Cryptosporidium* upper-bound dose-resposne results presented.

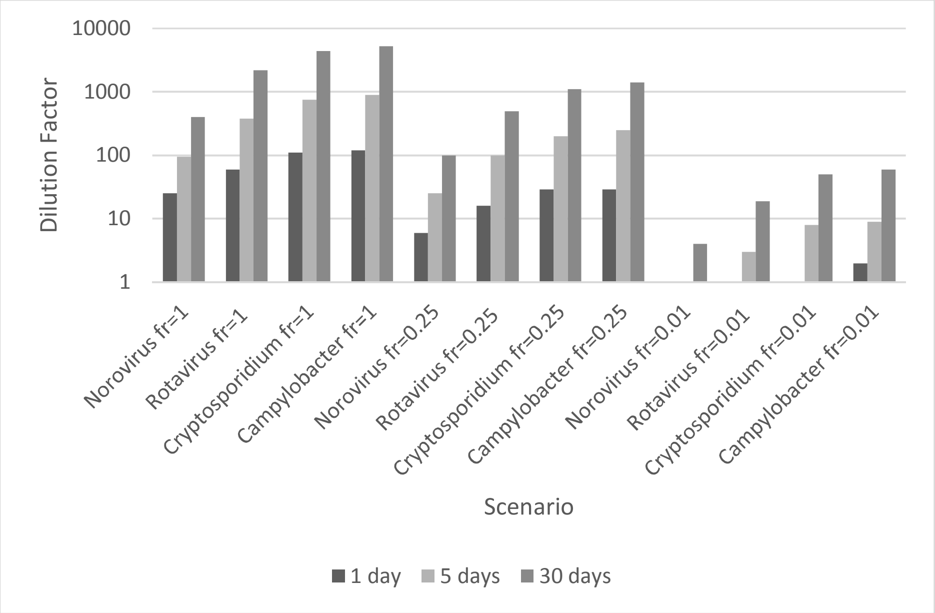

Figure S9. Safe dilution factor of intrusion of non-potable reclaimed wastewater (generated by 4,500-person office collection and treated for indoor reuse) to potable water supply that results in a 95th percentile annual risk of infection from non-potable indoor reuse equivalent to 10^{-4} ppy. [2].

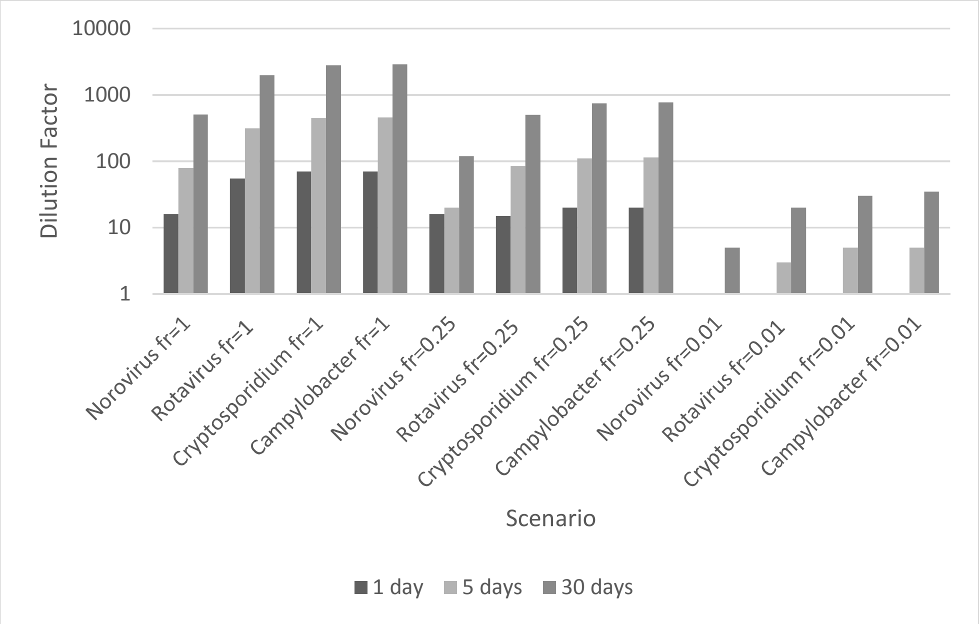

Figure S10. Safe dilution factor of intrusion of non-potable reclaimed greywater (generated by 40,000-person residential collection and treated for indoor reuse) to potable water supply that results in a 95th percentile annual risk of infection from non-potable indoor reuse equivalent to 10^{-4} ppy. [2.]

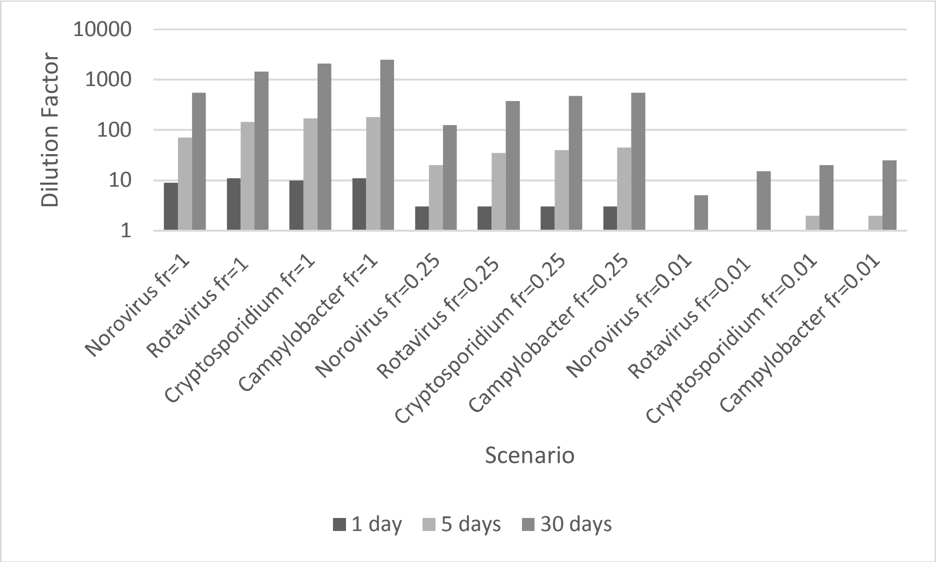

Figure S11. Safe dilution factor of intrusion of non-potable reclaimed greywater (generated by 1,000-person residential collection and treated for indoor reuse) to potable water supply that results in a 95th percentile annual risk of infection from non-potable indoor reuse equivalent to 10^{-4} ppy. [2.]

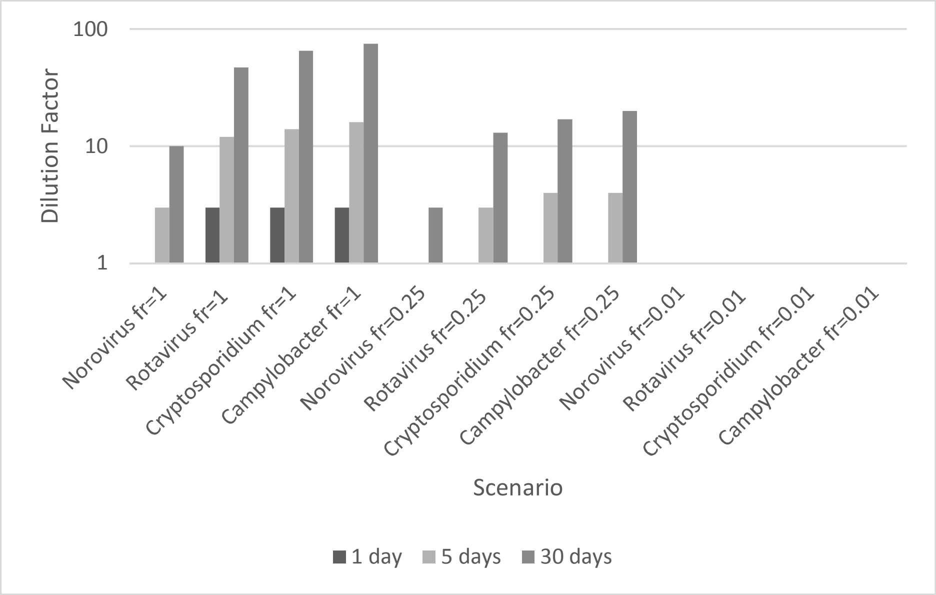

Figure S12. Safe dilution factor of intrusion of non-potable reclaimed greywater (generated by 4,500-person office collection and treated for indoor reuse) to potable water supply that results in a 95th percentile annual risk of infection from non-potable indoor reuse equivalent to 10^{-4} ppy. [2.]

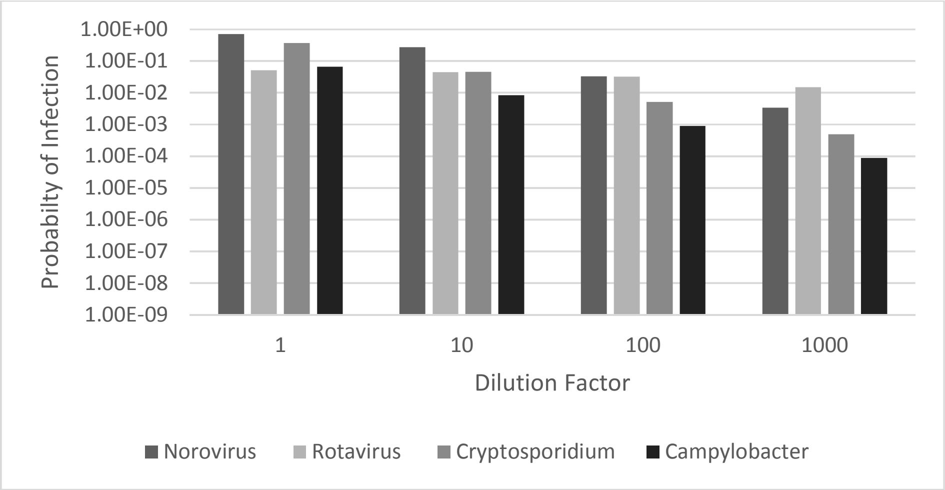

Figure S13. 95th percentile event probability of infection for ingestion of domestic, non-potable reclaimed wastewater contaminated by domestic wastewater generated by a 40,000-person residential collection system.[1] .

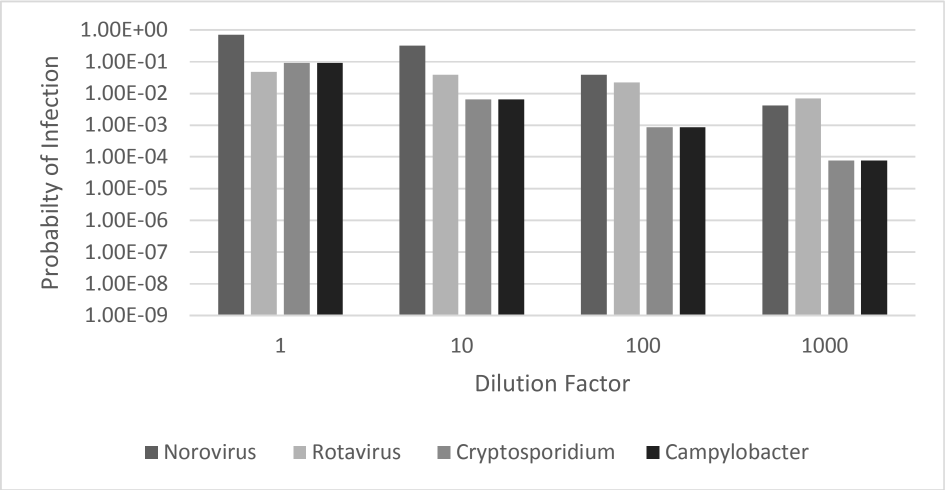

Figure S14. 95th percentile event probability of infection for ingestion of domestic, non-potable reclaimed wastewater contaminated by domestic wastewater generated by a 1,000-person residential collection system.[1] .

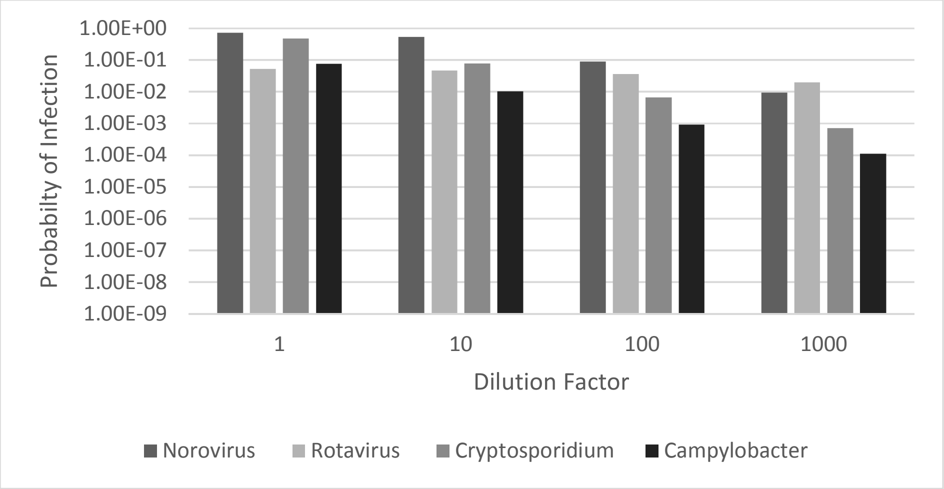

Figure S15. 95th percentile event probability of infection for ingestion of domestic, non-potable reclaimed wastewater contaminated by domestic wastewater generated by a 4,500-person office collection system.[1] .

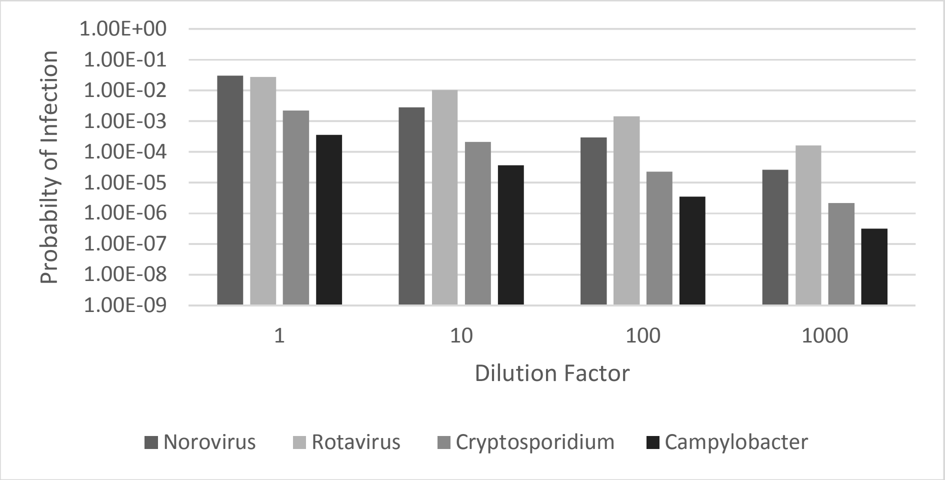

Figure S16. 95th percentile event probability of infection for ingestion of domestic, non-potable reclaimed greywater contaminated by domestic greywater generated by a 40,000-person residential collection system.[1] .

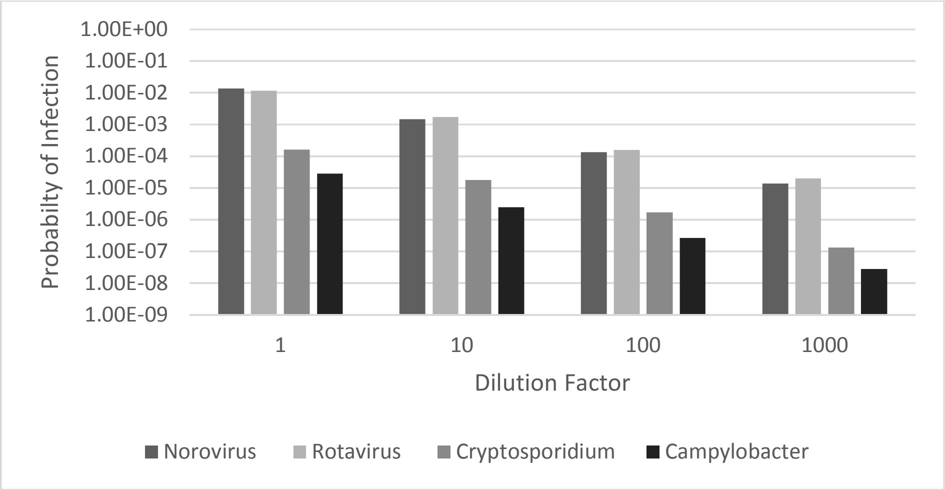

Figure S17. 95th percentile event probability of infection for ingestion of domestic, non-potable reclaimed greywater contaminated by domestic greywater generated by a 1,000-person residential collection system.[1] .

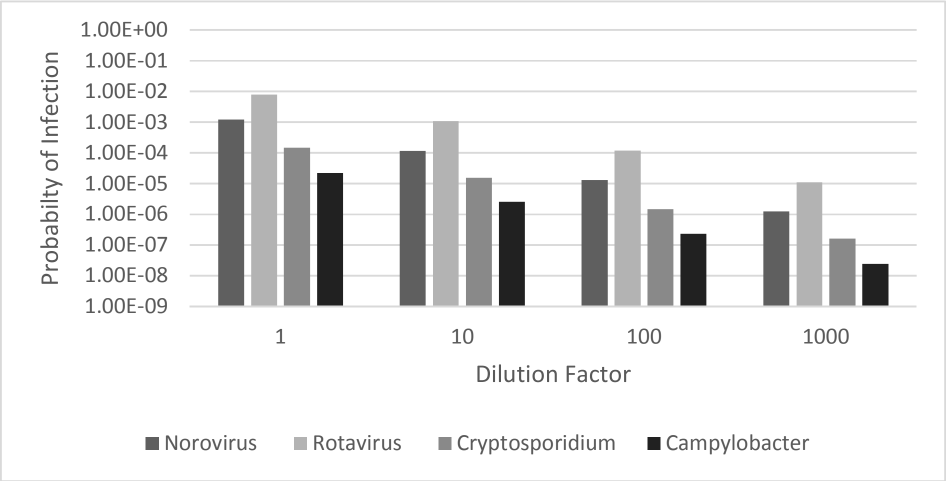

Figure S18. 95th percentile event probability of infection for ingestion of domestic, non-potable reclaimed greywater contaminated by domestic greywater generated by a 4,500-person office collection system.[1.]

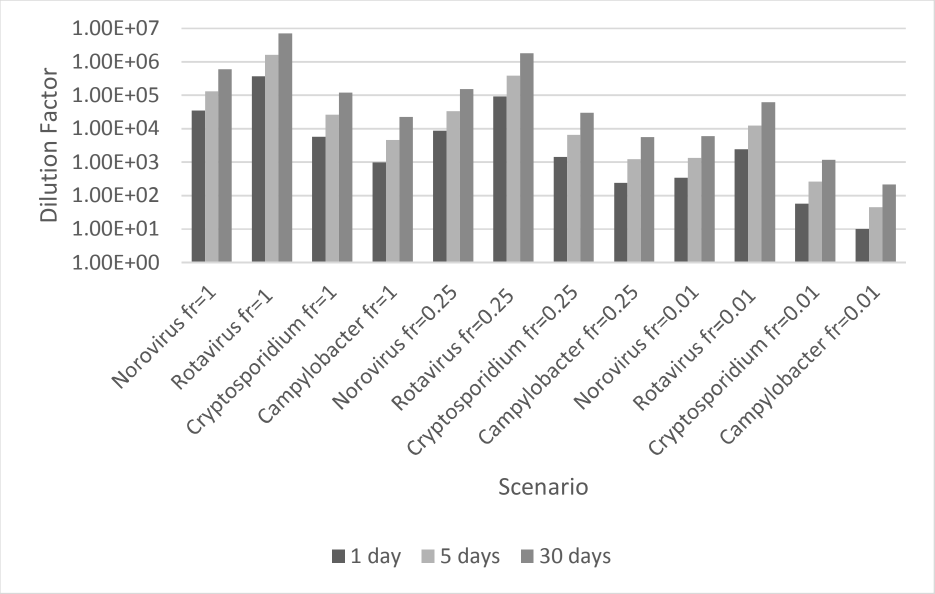

Figure S19. Safe dilution factor of intrusion of residential wastewater (generated by 40,000-person collection) to non-potable reclaimed water supply that results in a 95th percentile annual risk of infection from non-potable domestic, indoor reuse equivalent to the benchmark (10^{-4} ppy).[2.]

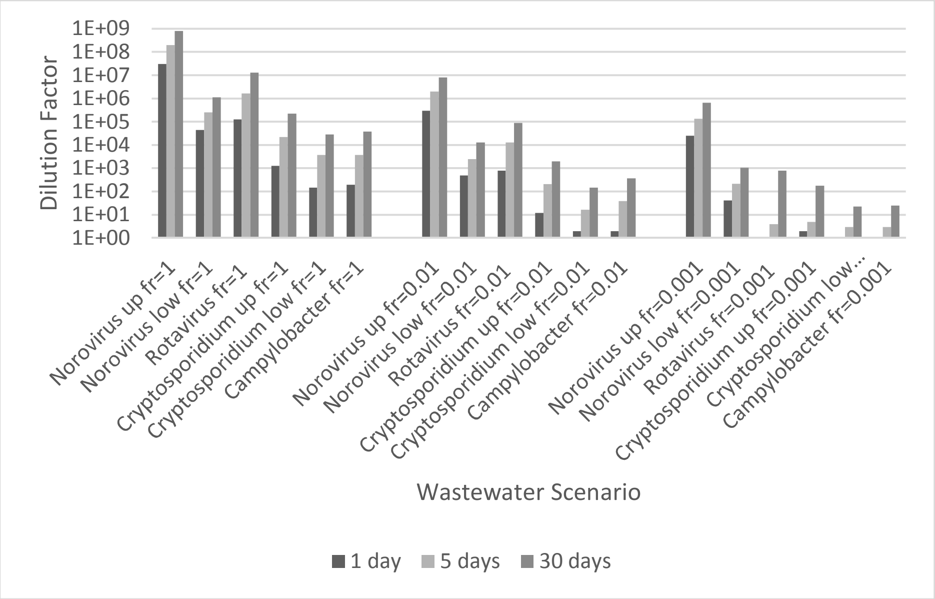

Figure S20. Safe dilution factor of intrusion of residential wastewater (generated by 1,000-person collection) to non-potable reclaimed water supply that results in a 95th percentile annual risk of infection from non-potable domestic, indoor reuse equivalent to the benchmark (10^{-4} ppy). [2.]

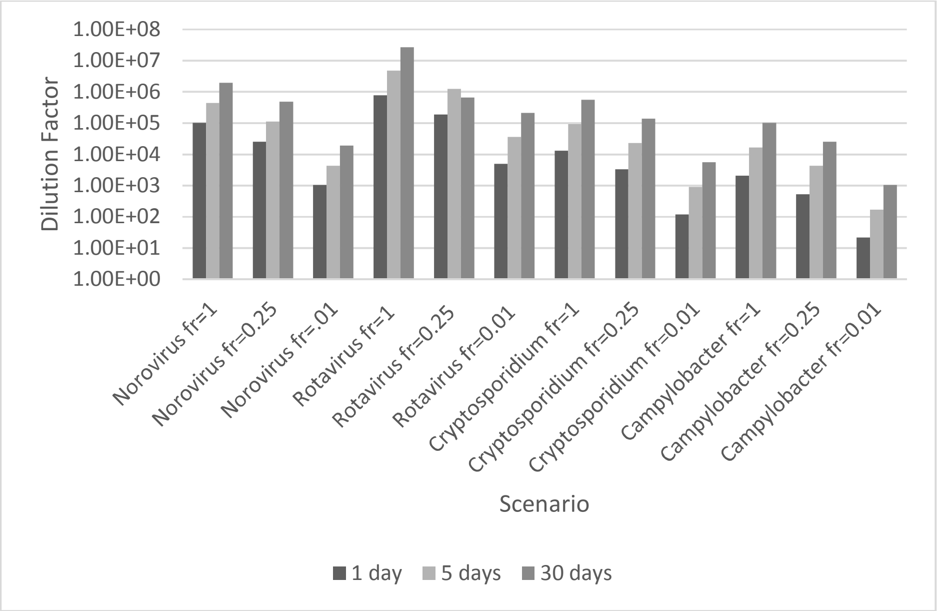

Figure S21. Safe dilution factor of intrusion of office wastewater (generated by 4,500-person collection) to non-potable reclaimed water supply that results in a 95th percentile annual risk of infection from non-potable domestic, indoor reuse equivalent to the benchmark (10^{-4} ppy). [2.]

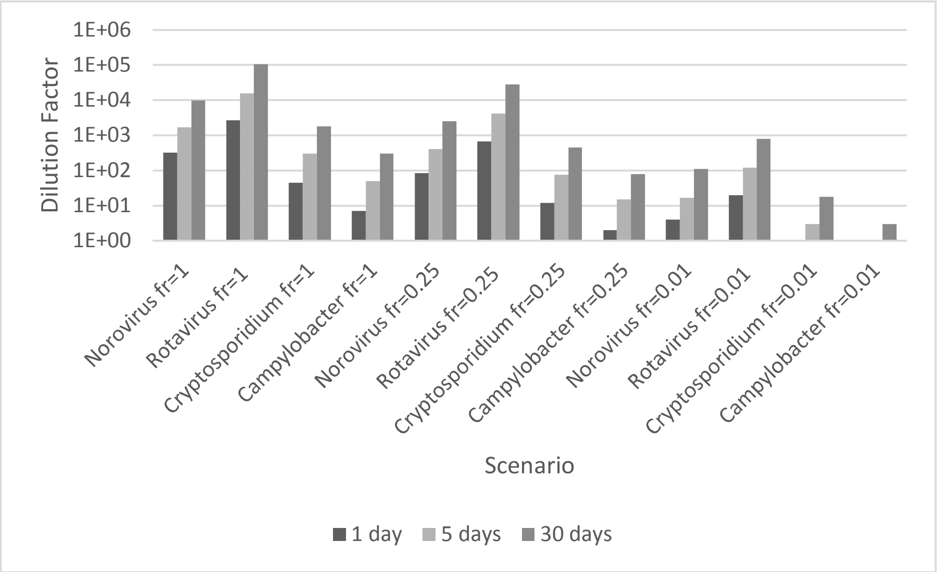

Figure S22. Safe dilution factor of intrusion of residential greywater (generated by 40,000-person collection) to non-potable water supply that results in a 95th percentile annual risk of infection from non-potable domestic, indoor reuse equivalent to the benchmark (10^{-4} ppy). [2] .

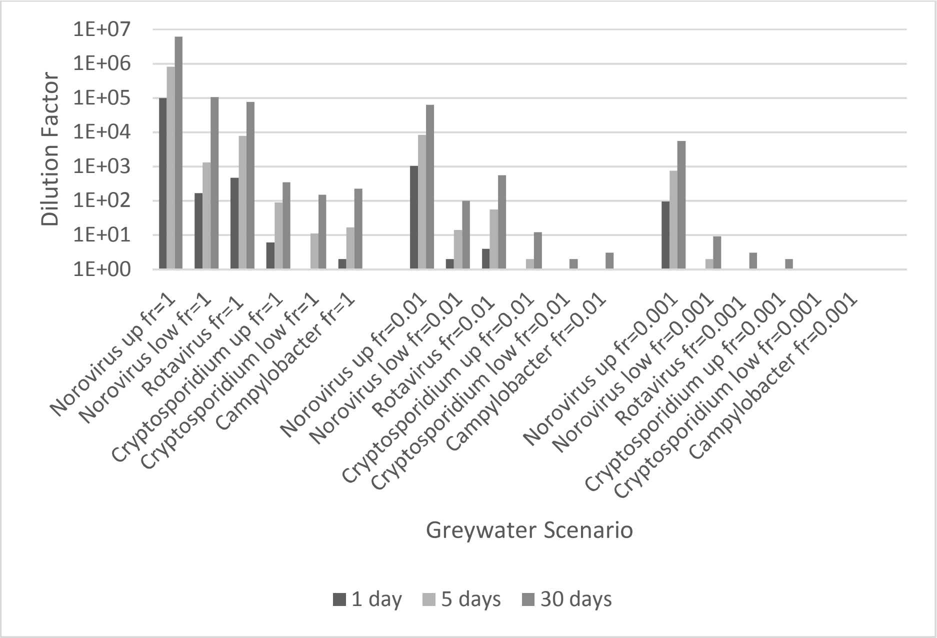

Figure S23. Safe dilution factor of intrusion of residential greywater (generated by 1,000-person collection) to non-potable water supply that results in a 95th percentile annual risk of infection from non-potable domestic, indoor reuse equivalent to the benchmark (10^{-4} ppy). [2]

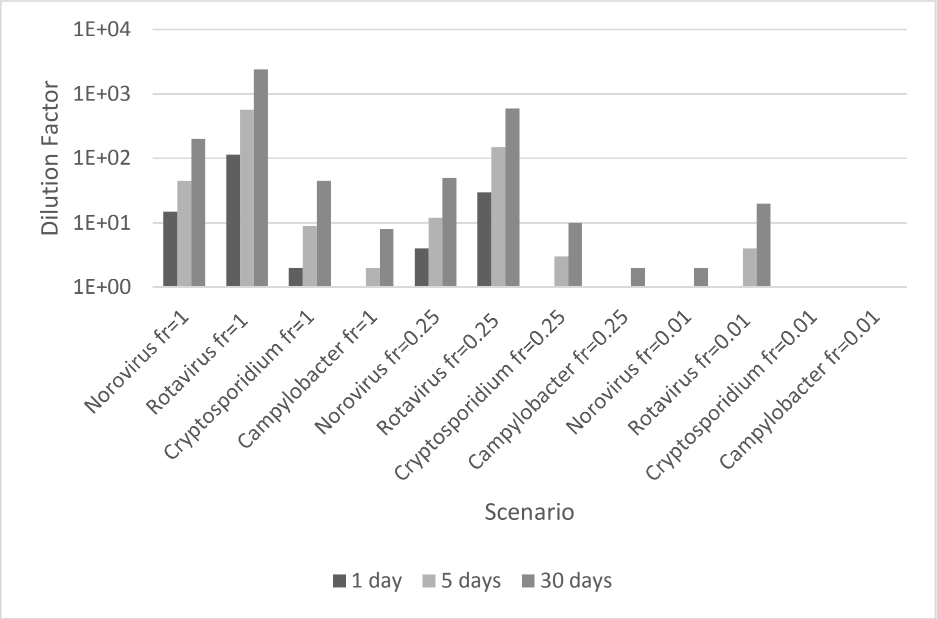

Figure S24. Safe dilution factor of intrusion of residential greywater (generated by 4,500-person collection) to non-potable water supply that results in a 95th percentile annual risk of infection from non-potable domestic, indoor reuse equivalent to the benchmark (10^{-4} ppy).[2.]
